# The Complement System: A Powerful Modulator and Effector of Astrocyte Function in the Healthy and Diseased Central Nervous System

**DOI:** 10.3390/cells10071812

**Published:** 2021-07-17

**Authors:** Marcela Pekna, Milos Pekny

**Affiliations:** 1 Laboratory of Regenerative Neuroimmunology, Center for Brain Repair, Department of Clinical Neuroscience, Institute of Neuroscience and Physiology, Sahlgrenska Academy at the University of Gothenburg, 40530 Gothenburg, Sweden; 2 Laboratory of Astrocyte Biology and CNS Regeneration, Center for Brain Repair, Department of Clinical Neuroscience, Institute of Neuroscience and Physiology, Sahlgrenska Academy at the University of Gothenburg, 40530 Gothenburg, Sweden; milos.pekny@neuro.gu.se; 3 Florey Institute of Neuroscience and Mental Health, Parkville, Melbourne 3010, Australia; 4 School of Medicine and Public Health, University of Newcastle, Newcastle 2308, Australia

**Keywords:** astrocytes, the complement system, C3, reactive astrocytes, reactive gliosis, neurodegeneration, neural plasticity

## Abstract

The complement system, an effector arm of the innate immune system that plays a critical role in tissue inflammation, the elimination of pathogens and the clearance of dead cells and cell debris, has emerged as a regulator of many processes in the central nervous system, including neural cell genesis and migration, control of synapse number and function, and modulation of glial cell responses. Complement dysfunction has also been put forward as a major contributor to neurological disease. Astrocytes are neuroectoderm-derived glial cells that maintain water and ionic homeostasis, and control cerebral blood flow and multiple aspects of neuronal functioning. By virtue of their expression of soluble as well as membrane-bound complement proteins and receptors, astrocytes are able to both send and receive complement-related signals. Here we review the current understanding of the multiple functions of the complement system in the central nervous system as they pertain to the modulation of astrocyte activity, and how astrocytes use the complement system to affect their environment in the healthy brain and in the context of neurological disease.

## 1. Introduction

By virtue of its ability to sense and rapidly respond to diverse danger signals, the complement system plays an essential role in the innate immune responses and constitutes the first line of defense against pathogens such as bacteria, viruses, mycoplasma, fungi, and protozoa. Whereas the functions of the complement system in the elimination of pathogens, antigen-antibody complexes, dead cells and tissue debris, and their involvement in the regulation of the responses mediated by antibodies and cells of the adaptive immune system have been well recognized for many decades, the homeostatic and non-immune tasks of the complement proteins only recently began to be unraveled. Liver cells are the main source of complement proteins in the blood, but complement proteins are also produced locally in many tissues including the central nervous system (CNS). There is an exponentially growing amount of evidence for the role of the complement system in the CNS development [1,2,3,4,5,6,7,8], maintenance and restoration of CNS homeostasis [9,10], and in the regulation of neural plasticity [11,12,13,14]. However, the complement system is also an important driver of age-related synapse loss and cognitive decline [15] and plays a principal role in the neurodegeneration processes [16,17,18,19,20,21,22]. Astrocytes are a major source of the complement system proteins, particularly of the third complement component (C3), and they stand out as a very prominent player or effector in many of the complement-mediated processes. Through a number of complement receptors expressed on their cell membrane, astrocytes are also well equipped to sense the level of complement activation in their vicinity, as well as receive and respond to complement-derived signals sent by other cells.

## 2. The Complement System

The complement system consists of more than 50 secreted and cell membrane-bound proteins that exert their functions through highly ordered interactions. Liver cells are the main source of complement proteins in the blood. Complement proteins are also produced locally in many tissues including the CNS. The very powerful and potentially deleterious effector functions of the complement system place high demands on its control. The strict control of the complement system is achieved at two levels: activation of inactive precursors and inactivation of the active components through degradation. Many of the secreted complement proteins are produced as inactive precursors and need to be activated through conformational changes induced, for example, by binding to the right target, such as the pathogen-associated molecular patterns or antigen-antibody complex, or through proteolytic cleavage. The complement system also includes a number of fluid-phase and membrane-bound regulatory proteins that protect healthy host cells and ensure that the activation and effector functions of the active proteins and their fragments are limited both in space and in time.

### 2.1. Activation of the Complement System

**Figure 1:**
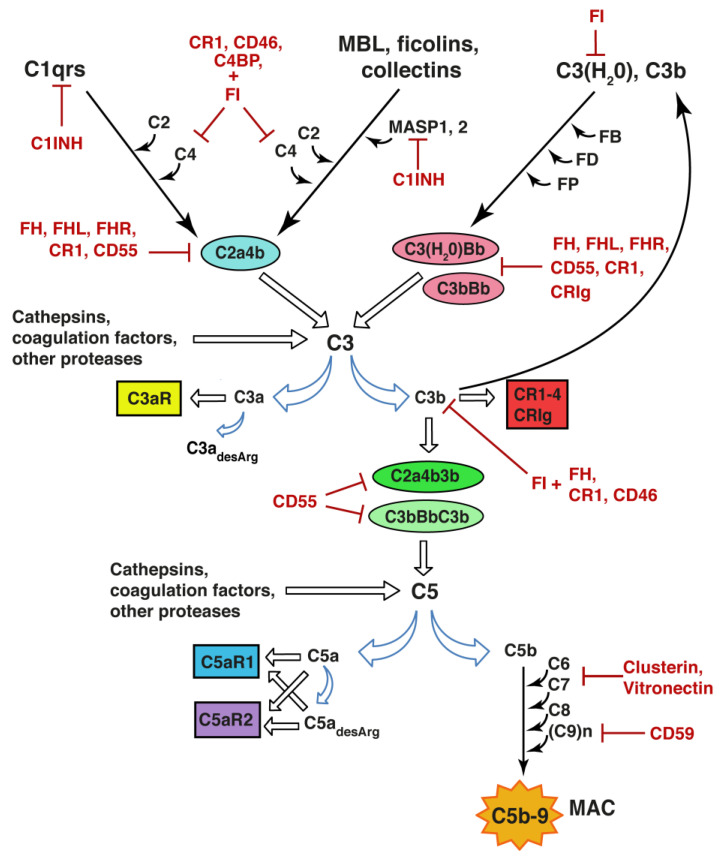
Complement system activation. Upon binding to a target structure, e.g., antigen-antibody complex or as yet unidentified synaptic component, C1q triggers the activation of the classical pathway leading to formation of the C2a4b convertase and the proteolytic activation of C3, opsonization of the target with C3b, and release of C3a. Recognition of microbial sugar moieties by ficolins, collectins or mannan-binding lectin (MBL) in complex with mannan-binding lectin-associated serine protease 1 and 2 (MASP1, 2) initiates the lectin pathway. The alternative pathway is activated when spontaneous hydrolysis of the internal thioester bond in C3 leads to the conformational change of the C3 molecule resulting in C3(H_2_O) with the ability to bind factor B (FB). C3(H_2_O) or C3b bound FB is cleaved by factor D (FD) to generate the C3(H_2_O)Bb and C3bBb convertase, respectively. These enzymatic complexes are stabilized by properdin (FP). Through binding of C3b, the C3 convertases acquire the ability to cleave C5 to form C5a and C5b. C3 and C5 can also be cleaved by cathepsins, several coagulation factors, and other proteases. C5b recruits C6, C7, C8, and multiple C9 molecules to form C5b-9, also called membrane attack complex (MAC) on the target membrane. C3a and C5a exert their effects through the C3aR1, C5aR1 and C5aR2 receptors. Removal of the C-terminal arginine by carboxypeptidases generates C3a_desArg_ and C5a_desArg_. C3b and some of its degradation fragments can bind to complement receptors (CR) 1–4 and CRIg. At multiple levels, the activation of the complement activation is negatively regulated by inhibitory enzymes and their cofactors. C1INH: C1 inhibitor; C4BP: C4 binding protein; FH: factor H; FHL: factor H-like protein; FHR: factor H-related protein; FI: factor I.

There are three canonical pathways through which the complement system can be activated: the classical pathway, the alternative pathway, and the lectin pathway. The prototypic trigger of the classical pathway is the binding of the C1 complex to the Fc moieties of antigen-bound IgG or IgM. The C1 complex consists of C1q, which serves the role of a pattern recognition molecule, associated with a tetramer of two each of the serine proteases C1r and C1s. Other ligands for the complement component C1q include molecular patterns on certain pathogens (such as Gram-positive and Gram-negative bacteria and some viruses), the C-reactive protein-phosphocholine complex, pentraxin-3, serum amyloid P component, beta-amyloid fibrils, DNA, mitochondrial membranes, as well as different targets on apoptotic cells such as phosphoserine, histones and annexins (reviewed by e.g., [23,24]). Binding of C1q to a ligand induces autoactivation of C1r followed by sequential proteolytic activation of C1s, C4 and C2, which results in the formation of activator-bound C3 convertase (C4b2a) of the classical pathway, an enzymatic complex that cleaves C3 into C3a and C3b. C3a is released into the fluid-phase and binds to the C3a receptor (C3aR) or is rapidly inactivated by carboxypeptidases that remove its C-terminal arginine residue. C3b becomes covalently bound to the activator via exposed internal thioester bond, thus irreversibly tagging and opsonizing the activator for recognition by cells expressing complement receptors (CR) 1–4 and CRIg, Figure 1.

The pattern recognition molecules of the lectin pathway are the collectins mannan-binding lectin and collectin-LK and ficolins H, M, and L, which recognize the sugar moieties and acetyl groups of glycoproteins and glycolipids on bacteria, viruses, fungi, and protozoa, but also host organelles, mitochondria and natural IgM bound to neo-epitopes exposed on apoptotic and necrotic host cells. Similar to the C1 complex of the classical pathway, binding of collectins or ficolins to an activator leads to the autoactivation and proteolytic activation of the serine proteases they are associated with, namely mannan-binding lectin-associated serine protease 1 and 2, followed by activation of C2 and C4 and formation of activator-bound C3 convertase C4b2a. Activation of the classical and lectin pathways thus leads to the covalent binding of C3b to the activator surface, Figure 1.

The C3 convertase of the alternative pathway is formed through the recruitment of factor B to an activator-bound C3b; factor B in complex with C3b is subsequently cleaved by factor D, thus generating the C3 convertase C3bBb. The C3bBb complex then generates multiple copies of C3b and serves as a very powerful amplifier of activation triggered by the classical and lectin pathways. In addition to its generation through the activator-bound C4b2a and C3bBb convertases, C3b can be produced by the fluid-phase C3 convertase C3(H_2_O)Bb. C3(H_2_O) is constitutively generated by a slow-rate hydrolysis of the internal thioester bond of C3, which leads to the conformational change of the C3 molecule, acquisition of C3b properties, and binding of factor B. While the C3(H_2_O) activity is proteolytically inhibited by factor I, the half-life of the C3 convertases of the alternative pathway is increased by properdin, which acts as a C3 convertase-stabilizing factor, Figure 1.

Through the recruitment of C3b, the activator-bound C3 convertases acquire the ability to cleave C5 into C5a and C5b, and thus initiate the terminal pathway of complement activation. While C5a is released into the fluid-phase and exerts its actions through binding to cell membrane receptors C5aR1 and C5aR2, C5b binds to the activator surface and together with C6, C7, C8 and multiple C9 molecules becomes part of the membrane attack complex that forms a pore in the cell membrane and can lead to the lysis of the target cells or bacteria. In sublytic amounts, the membrane attack complex can modulate inflammation-associated functions of the host cell.

In addition to the convertases mentioned above, C3 and C5 can be proteolytically activated directly by mannan-binding lectin-associated serine protease 1 [25], non-complement proteases such as neutrophil elastase, cathepsins [26,27], granulocyte neutral proteases [28], lysosomal enzymes, kallikrein, as well as coagulation factors XIa, Xa, IXa, thrombin, and plasmin [29,30], Figure 1.

### 2.2. Regulators of the Complement System

As mentioned above, the constitutive mode of activation together with the huge amplification power of the alternative pathway pose potential danger to the host cells and thus demand strict spatiotemporal control of the active components, convertases, as well as the membrane attack complex. The protection of host cells from the bystander effect of C3b deposition is achieved by fluid-phase regulators such as factor H, factor H-like protein, complement factor H-related proteins 2 and 4, and C4-binding protein, which all facilitate the decay of the C3 convertases. In addition, factor I is a serine protease that degrades cofactor bound C3b and C4b, C1-inhibitor inhibits serine proteases including those with complement-activating properties. Membrane-bound CR1 (CD35) and decay accelerating factor (CD55) facilitate the decay of the C3 convertases. CD46 and CR1 act as cofactors for factor I-mediated proteolytic degradation of C4b, C3b and their fragments. The formation of membrane attack complex on host cells is inhibited by fluid-phase clusterin and vitronectin, and the membrane-bound CD59. The biological activity and potency of C3a and C5a is controlled through the removal of C-terminal arginine residue by carboxypeptidases generating C3a_desArg_ and C5a_desArg_, respectively, [24]), Figure 1.

### 2.3. The Complement System Receptors

The five currently known CRs all bind C3b and/or its degradation products iC3b and C3d. CR1 binds C3b, C4b, as well as iC3b and C3d. It serves to remove antigen-antibody complexes, promote phagocytosis, and to capture complement-opsonized antigens for B cell stimulation by antigen presenting cells, the latter a function shared with CR2 (CD21). The heterodimeric receptors CR3 (CB11b/CD18) and CR4 (CD11c/CD18) are phagocytic receptors that bind iC3b. The fifth CR, CRIg, is involved in the clearance of C3b and iC3b tagged pathogens by the Kupffer cells in the liver. CRIg also inhibits the C3bBb convertase.

C3a and C5a, the smaller fragments generated through the proteolytic activation of C3 and C5, respectively, exert their functions through their canonical G-protein coupled receptors C3aR and C5aR1. C3aR and C5aR1 are expressed on endothelial, smooth muscle and myeloid cells, and signaling through these receptors has long been known to increase vascular permeability, stimulate smooth muscle contraction, and leukocyte chemotaxis, as well as activate myeloid cells such as neutrophils, monocytes/macrophages, basophils, and platelets [31]. While C3a_desArg_ no longer binds to C3aR, C5a_desArg_ binds to C5aR1 but does not activate receptor signaling. Both C5a and C5a_desArg_ also bind to C5aR2. Although unable to couple to G-proteins, C5aR2 can function as a positive modulator for responses induced by C3aR and C5aR [32] as well as other signaling pathways [33].

### 2.4. The Intracellular Complement System

Many types of human cells, including T cells, B cells, monocytes, neutrophils, fibroblasts, and airway epithelial cells take up, store, and intracellularly activate C3 through a convertase-independent mechanism [26,34,35]. Intracellular complement, in particular C3, and C3a and C3b generated from C3 by cathepsin L, is essential for the development of T cell-mediated responses [26], promotes cell survival [34,35,36] and modulates intracellular metabolism [37]. Notably, even cells that are not able to produce C3 have been shown to internalize C3 in its hydrolytic form C3(H_2_O) and use it as the source of C3a, although the specific receptor involved in this process remains to be identified [38].

## 3. Astrocytes, Astrocyte Activation and Reactive Gliosis

Astrocytes are glial cell of neuroectodermal origin that have many functions in both healthy and diseased CNS [39,40,41]. Through thousands of fine cellular processes, astrocytes are in contact with neuronal synapses and form end-feet wrapping around blood capillaries, constituting a key component of the blood–brain barrier [40,42,43]. Astrocytes play a major role in the maintenance of the CNS homeostasis, control and support of neurons, neurotransmitter recycling, control of blood flow, and the induction, functional control, and removal of neuronal synapses [44,45,46,47,48,49,50,51]. Astrocytes are also an essential component of the glymphatic system, a brain-wide fluid transport pathway that supports the clearance of potentially harmful metabolic or protein waste products, such as amyloid-β, through the rapid and circadian rhythm-dependent exchange of cerebrospinal fluid and interstitial fluid along perivascular pathways [52,53].

Changes in CNS homeostasis, aging, as well as various kinds of pathological conditions, including acute and chronic injury and infection, trigger a broad range of general and context-specific astrocyte responses [54,55,56]. These responses span from astrocyte activation, induced by physiological signals and physiological alterations of CNS homeostasis, to disease and injury-associated astrocyte reactivity [41,55]. Reactive astrocytes are key components of reactive gliosis, which also includes reactive microglia [54,57,58,59]. Astrocyte reactivity is not an on–off response, but rather a continuum of multiple states [39,56] with both general and disease-specific components, with astrocytes in the same tissue and disease condition exhibiting substantial heterogeneity [60,61]. The most severe form of reactive gliosis is the glial scar that seals the penetrating lesion from the surrounding healthy nervous tissue [39,62]. The functions of reactive astrocytes are disease-specific and often even disease stage-dependent, encompassing neuroprotection and adaptive plasticity-promoting properties on one end and neurotoxicity and malfunction on the other end of their phenotypic spectrum [41].

The JAK/STAT3 signaling pathway is an important switch controlling a number of molecular and functional changes in reactive astrocytes. Signaling molecules, such as transforming growth factor (TGF)α, ciliary neurotrophic factor (CNTF), interleukin (IL)-6, leukemia inhibitory factor (LIF), or oncostatin M have been shown to trigger astrocyte activation [63,64,65,66,67,68]. These signaling events lead to changes in gene expression, metabolism, cell morphology and cell proliferation [39,69]. Indeed, reactive astrocytes are characterized by hypertrophy of their cellular processes and exhibit changes in the expression of many genes including the up-regulation of glial fibrillary acidic protein (GFAP), the main component of astrocyte cytoplasmic intermediate filaments (also known as nanofilaments) [39,70,71]. Astrocyte cytoplasmic nanofilaments are composed of GFAP, vimentin, and in some cases also nestin and synemin [71,72,73,74,75]. Cytoplasmic nanofilaments are highly dynamic structures that are involved in cell signaling and cell migration, determine cellular viscoelastic properties, and act as a signaling platform regulating cell stress responses [39,73,76,77,78,79].

Many of the complement proteins, including the proteins involved in complement activation, complement receptors as well as regulators (CD46, CD55, CD59), are expressed by astrocytes. As the roles of complement in CNS responses to infection have been discussed elsewhere, we will focus on the novel, non-canonical and modulatory functions of the complement system with implications for astrocytes.

## 4. The Complement System: A Modulator of Astrocyte Functions

**Figure 2:**
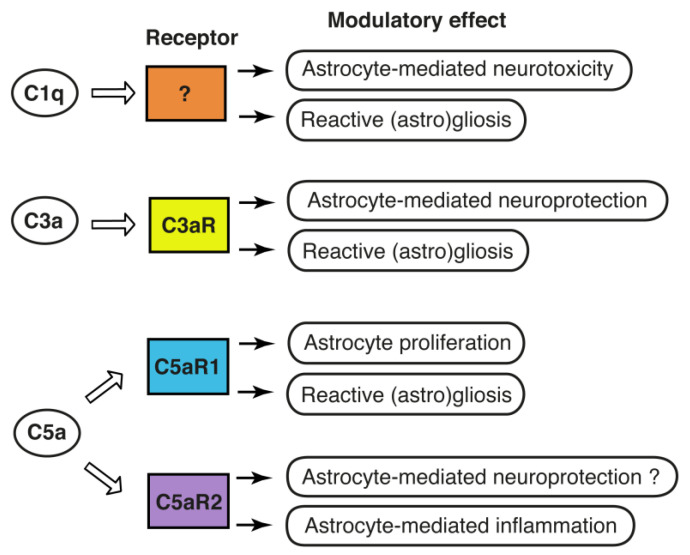
Complement is a modulator of astrocyte function: known and putative effects of the complement components C1q, C3 and C5 on astrocytes. While there are several potential candidates, the specific receptor through which C1q exerts its modulatory effects on astrocytes is currently unknown. C3a and C5a exert their modulatory effects through C3aR, C5aR1 and C5aR2.

The reciprocal signaling between astrocytes and microglia, the resident immune cells of the CNS, appears to play a critical role in determining the reactive astrocyte phenotype. Astrocytes express C3aR [80,81,82], C5aR1 [83,84,85], C5aR2 [86], and CR3 [87], as well as Megf10, a phagocytic receptor that can also bind C1q [88]. gC1qR, a receptor for the globular domain of C1q, with a potential role in phagocytosis, and cC1qR/calreticulin which binds the collagen domain of C1q [89], were reported to be expressed on human astrocytes [90] and on rat astrocytes [91], respectively. CD91/low-density lipoprotein receptor-related protein, is a multi-scavenger receptor that was shown to directly interact with C1q for clearance of C1q and C1q-bound material [92], and is expressed by multiple cell types including astrocytes [93]. CD93 is another C1q binding phagocytic receptor that is expressed by neural stem cells and neurons [94]. CD93 negatively regulates astrogenesis in the developing brain but does not seem to be expressed by mature astrocytes [94]. Human astrocytes were reported to also express CR1 and CR2 [95,96]. Given their broad expression of receptors for complement proteins and their activation products, it is therefore not surprising that the complement system has emerged as an important factor in the cross-talk between microglia and astrocytes, Figure 2.

Microglia-derived cytokines such as TNFα, IL-1β and IL-6 were shown to transform astrocytes into neuroprotective phenotype [97], whereas C1q has been put forward as one of the signaling molecules needed for the induction of the so called neurotoxic astrocyte by microglia [98] and an Alzheimer’s disease-specific modulator of the cellular cross-talk between microglia and astrocytes [99]. However, the astrocyte receptor needed for C1q to exhibit this effect is not known. One possible candidate is Megf10, which is predominantly expressed by astrocytes [87]. Astrocyte expression of Megf10 and CR3 is increased with age [100] as is the microglial expression of C1q [101]. Indeed, binding of C1q to Megf10 is required for normal clearance of apoptotic cells by astrocytes [88], and Megf10 is critical for neuronal activity-dependent synapse remodeling by astrocytes [102]. Other candidates worth investigating in this context include cC1qR, gC1qR and CD91.

Using human pluripotent stem cell-derived tri-culture system containing neurons, astrocytes and microglia, a recent study demonstrated that, at least in the context of human Alzheimer’s disease, C3 secreted by astrocytes is required for increased C3 release by microglia [99]. Further, this study showed that the ability of microglia to clear C1q-complexes is impaired when microglia are cultured with C3-deficient compared to wild-type astrocytes [99]. These findings identified C3 as a critical factor in reciprocal signaling between microglia and astrocytes; however, the C3 or C3-derived fragment-binding entity on microglia is currently unknown. In light of its high expression by microglia, C3aR is clearly a candidate receptor to be investigated in this context. Indeed, cross-talk between astrocyte secreted C3 and microglial C3aR was shown to regulate phagocytosis of amyloid-β by cultured primary microglia [103].

Astrocytes express C3aR [80,81,82] and respond to C3a by changes in intracellular signaling [84]. In the unchallenged CNS, the source of C3a is conceivably C3 produced by the glial cells. In the acutely injured brain, C3-C3a can originate from astrocytes and microglia, as well as from the systemic circulation via leakage of C3 to the brain parenchyma through the dysfunctional blood-brain barrier. The exact mechanisms of complement activation and C3a generation in the naive and injured brain warrant further investigation. Insults such as brain ischemia increase astrocyte expression of C3aR [82,104,105], and there is experimental evidence for the neuroprotective effects of C3aR signaling in astrocytes. Astrocytes respond to C3a by the expression of cytokines such as interleukin (IL)-6, IL-8, and nerve growth factor (NGF) [83,106,107]. C3a promotes astrocyte survival after ischemic stress by inhibiting ERK signaling-mediated apoptotic pathway and caspase-3 cleavage [82]. C3a is neuroprotective against excitotoxicity, but only when neurons are co-cultured with astrocytes [108]. In vivo studies provide further support for the beneficial effects of C3a/C3aR after brain injury and the involvement of astrocytes therein. Transgenic mice expressing C3a under the control of GFAP promoter, i.e., in astrocytes, were protected against brain tissue loss due to neonatal hypoxic-ischemic brain injury [109], a major cause of long-term neurological sequelae of birth complications such as intellectual disability, epilepsy, and cerebral palsy [110]. Memory impairment due to neonatal hypoxic-ischemic injury was mitigated by intraventricular administration of C3a in wild-type mice but not in mice lacking C3aR [109]. Intranasal treatment with C3a reduced reactive gliosis in mice with neonatal hypoxic-ischemic injury and improved long-term cognitive function [111]. In the adult brain, signaling through neuronal C3aR modulates dendritic morphology and the function of synapses [17], and stimulates neural plasticity after ischemic stroke [13]. However, given the expression of C3aR on astrocytes and microglia, C3a may also exert its effects indirectly through glial C3aR, and the balance between neuronal and glial C3aR signaling may be developmentally regulated, Figure 2. Regardless of its cellular expression, C3aR appears to be an attractive target for pharmacological manipulation to improve outcome after ischemic injury to both the adult and the immature brain.

While contributing to tissue injury in the acute period, C5a/C5aR1 signaling is required for optimal protective and reparatory astrocyte responses and for the formation of glial scar in the post-acute phase [112]. In a rodent model of amyotrophic lateral sclerosis, both the expression levels of C5aR1 and the number of C5aR1-expressing astrocytes increase in the spinal cord with disease progression [16]. Treatment with C5aR1 antagonist reduced astrocyte proliferation, slowed down the disease progression and extended survival [16]. C5aR2 has recently been shown to convey neuroprotection in a model of traumatic spinal cord injury [113]. C5aR2 knockdown increased the expression of nitric oxide synthase 2 and nuclear factor kappa B (NFκB) signaling in astrocytes, implicating C5aR2 as a negative regulator of inflammation [86]. The above neuroprotective functions of C5aR2 may therefore be astrocyte-mediated. However, as C5aR2 is expressed by astrocytes, neurons as well as microglia [86,87], the specific roles of C5aR2 in these cell-types and under different disease conditions remain to be addressed, Figure 2.

## 5. The Complement System: An Astrocyte Effector

**Figure 3:**
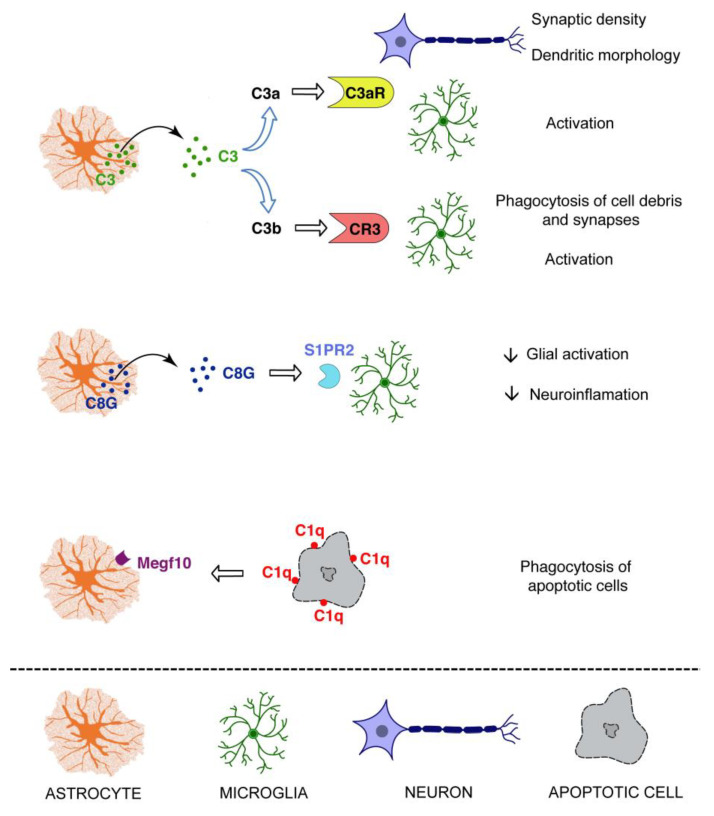
The complement system as an astrocyte effector. Astrocytes may exert some of their functions through the secretion of complement proteins, in particular C3 and the γ subunit of C8 (C8G), or through the phagocytosis of C1q coated targets. S1PR2: sphingosine-1-phosphate receptor 2.

In the developing CNS, astrocyte-derived TGFβ instructs neurons to tag weak synapses with C1q [114], which then leads to the activation of the classical complement pathway and synapse elimination via the recognition of synapse-bound C3b by microglial CR3, and its subsequent phagocytosis [7,8]. The C1q and CR3-dependent phagocytosis is also of critical importance for the clearance of neurodegenerative debris by microglia after injury [10], although the specific role of astrocytes in this process is less understood. As mentioned above, binding of C1q to a phagocytic receptor Megf10 is required for normal clearance of apoptotic cells by astrocytes [88]. Astrocyte Megf10 is also involved in developmental synaptic pruning and neuronal activity-dependent remodeling of synapses; however, the synapse engulfment by astrocytes is independent of C1q [8,102]. There is evidence pointing to the role of astrocyte-derived C3 and neuronal C3aR signaling in synaptic plasticity and maintaining normal dendritic extension [17]. However, exposure to amyloid-β activates astrocyte NFκB and triggers secretion of C3, which through subsequent excessive activation of C3aR on neurons seems to impair dendritic morphology and alter excitatory synaptic function through dysregulation of intraneuronal calcium [17]. Moreover, in the Alzheimer’s disease context, constitutive deficiency of C3 was protective against neurodegeneration and cognitive decline, despite higher amyloid-β plaque load [115]. Astrocyte-secreted C3 signaling through C3aR on microglia was proposed to contribute to tau-induced neurodegeneration by STAT3 activation leading to microglial reactivity [19]. Together with the evidence for the involvement of C3b in the loss of neuronal synapses [8,18], these results indeed point to the role of astrocyte derived C3 in neurodegeneration induced by amyloid-β. The C3–C3aR cross-talk between astrocytes and microglia drives astrocyte dysfunction, lesion development and functional impairment in a model of neuromyelitis optica [116], a rare neuroinflammatory demyelinating disease of the CNS characterized by the presence of autoantibodies against astrocyte water channel aquaporin 4, Figure 3.

In contrast, up-regulation of C3 in astrocytes was protective from neuronal damage and loss in the early stage of glaucoma [117]. In the same disease context, however, C1q, conceivably microglia-derived, was shown to play a deleterious role [118]. Thus, at least in some neurodegenerative conditions, C1q and C3 may have independent or even opposing roles. In the post-acute and chronic phase after ischemic stroke, C3a-C3aR signaling stimulates neurogenesis, the increase in the density of excitatory synapses, and the expression of GAP43, a marker of axonal sprouting and glial plasticity in the peri-infarct region [13,14]. Although the origin of C3/C3a in the peri-infarct parenchyma was not investigated, it is conceivable that astrocytes and microglia are two major sources of C3. In light of the growing evidence for the role of intracellular C3 in survival and metabolic reprogramming of other cell types [34,35,36,37], functions of C3 in astrocyte resilience [119] ought to be considered. The specific involvement of astrocyte C3 in different disease conditions and disease stages warrants further investigation, Figure 3.

A recent report pointed to a novel, complement activation-independent function of C8. The C8γ chain, one of the three subunits (α,β,γ) of C8, a component of the membrane attack complex, is present at high levels in the brain tissue, cerebrospinal fluid, and plasma of Alzheimer’s disease patients [120]. Importantly, C8γ is secreted by astrocytes, inhibits glial hyperactivation, neuroinflammation, and cognitive decline in acute and chronic animal models of Alzheimer’s disease, and exerts its anti-inflammatory effects via microglial sphingosine-1-phosphate receptor 2 [120], Figure 3.

## 6. The Complement System: A Marker of Astrocyte Phenotype?

Using transcriptomic data analysis, C3 was shown to be expressed by astrocytes isolated from brain tissue from several different acute and chronic neurodegenerative conditions and was put forward as a marker of what some authors referred to as the neurotoxic (A1) astrocytes [98]. However, C3 is also expressed in so called neuroprotective astrocytes (A2) [100] and microglia [99,121]. As detailed above, the exact functions of astrocyte C3 (secreted or intracellular) are not equivocal. They are complex, and context-dependent, and they are far from elucidated. For this and many other reasons, the misleading simplistic view and the binary division of reactive astrocytes into neuroprotective and neurotoxic were recently proposed to be abandoned, and this consensus view received a wide support from the astrocyte community [41]. The heterogeneity of reactive astrocytes, not least with regard to the expression of genes coding for complement proteins and receptors and their context-dependent functions, deserves further investigation.

## 7. Conclusions

Although the first report on the expression of complement proteins and receptors in astrocytes dates back more than 30 years [122], the full complexity of the multiple roles of complement in these cells is only now being appreciated. The scientific endeavor in search of novel functions of complement in the CNS is more active than ever. The rewards will come in the form of detailed understanding of the intricate cross-talk between neural cells and the roles of complement in the healthy CNS and a whole range of neurological diseases. Most likely they will also result in the identification of novel disease-specific strategies to reverse astrocyte dysfunction and modulate reactive gliosis for the treatment of neurodegenerative disorders and other pathological conditions affecting the CNS.
